# COVID-19 Highlights the Need for Inclusive Responses to Public Health Emergencies in Africa

**DOI:** 10.4269/ajtmh.20-1485

**Published:** 2020-12-15

**Authors:** Yusuff Adebayo Adebisi, Aniekan Ekpenyong, Blaise Ntacyabukura, Mat Lowe, Nafisat Dasola Jimoh, Toyyib Oladimeji Abdulkareem, Don Eliseo Lucero-Prisno

**Affiliations:** 1 Faculty of Pharmacy, University of Ibadan, Ibadan, Nigeria;; 2 Global Health Policy Unit, University of Edinburgh, Scotland, United Kingdom;; 3 Global Public Health Department, Karolinska Institutet, Stockholm, Sweden;; 4 Society for the Study of Women’s Health (SSWH), Old Yundum, Gambia;; 5 Federal University of Technology Minna, Minna, Nigeria;; 6 Institute of Health, Faculty of Education, Health and Wellbeing, University of Wolverhampton, Wolverhampton, United Kingdom;; 7 Department of Global Health and Development, London School of Hygiene and Tropical Medicine, London, United Kingdom

## Abstract

COVID-19 is a global public health emergency affecting many countries around the world. Although African governments and other stakeholders are making efforts to contain the pandemic, the outbreak continues to impact human rights and exacerbates inequalities and disparities that are already in existence. The concept of inclusive health focuses on good health and well-being for everyone, and this entails health services that are equitable, affordable, and efficacious. Creating equitable access to mainstream health and healthcare services and ensuring inclusive health responses remain a means of addressing health inequities and disparities. In this article, we argue on the need for inclusive responses to public health emergencies in Africa using COVID-19 as a case example. Africa’s response to public health emergencies needs to recognize that for every marginalized/vulnerable group, it is important to strategize to address their particular needs in such a way to surmount any barrier to the right to health. For Africa’s public health response to be more inclusive, we therefore need to be more strategic and proactive in reaching out to specific groups and to identify and address their needs. Strengthening the healthcare systems of African countries through increased political will, increased funding to health care, collaboration and cooperation among stakeholders, and effective leadership remains essential in ensuring inclusive responses to health emergencies.

## COMMENTARY

The COVID-19 pandemic continues to threaten public health systems around the world, and the African continent is not spared.^1–3^ The pandemic presents an unprecedented humanitarian crisis that has impacted health systems and disrupted the livelihood and overall well-being of people globally.^4,5^ Although the number of COVID-19 cases in Africa is currently lower than that of other regions of the world, these numbers are increasing gradually.^1^ As of November 14, 2020, 1,965,485 cases and 47,134 deaths have been reported in the African region.^6^ The pandemic has made evident weaknesses in responding to public health emergencies such as COVID-19, on the continent.^1^ Although African governments and other stakeholders continue to make efforts to contain the pandemic, there is more to be done to put the pandemic to an end. Nonetheless, the COVID-19 pandemic continues to impact human rights and exacerbates inequalities and disparities that are already in existence. Despite the efforts to address the COVID-19 pandemic on the continent, the marginalized and underrepresented groups have been reported to be left behind and discriminated against in the course of the COVID-19 responses.^7,8^ Creating equitable access to mainstream healthcare services and ensuring inclusive health responses serve as a means of addressing health inequities and disparities. In this article, we argue on the need for inclusive health response to public health emergencies in Africa using COVID-19 as a case example.

The concept of inclusive health focuses on good health and well-being for everyone; and this entails health services that are equitable, affordable, and efficacious.^9^ This concept also resonates with a rights-based approach to health including political, social, economic, scientific, and cultural actions that are geared toward advancing the cause of good health and well-being for all.^9^ With the emergence of the COVID-19 pandemic, it is increasingly important to ensure an inclusive health approach to health emergencies on the continent. It is now evident that it is not all about achieving “health for all” but an “inclusive approach” in meeting the health needs and responding to public health emergencies.^9^ In the context of the COVID-19 pandemic and other public health emergencies, health responses need to be genuinely empowering so that vulnerable, underrepresented, and marginalized “voices” are included in social discourse, policy, and the health response. Although most health policy documents may refer to “all people,” “for all,” “all citizens,” or “everybody,” they often end up privileging “some” over “all.”^9^ Vulnerable and marginalized populations usually have greater healthcare needs than others and are therefore more vulnerable to the impact of low-quality, inaccessible healthcare services as well as noninclusive uniform public health responses than others.^8^

In addressing public health issues, it is important to make it clear that no one is more important than the other, and the response efforts must not leave behind the most disadvantaged groups. The impact of public health response needs to be viewed from the lens of how it will affect the marginalized group and what can be done to prevent any form of inequity. Right to health is a human right, so the approach to responding to health emergencies needs to be able to ensure that right to equitable health is not infringed on and the outcome of the response should not weigh negatively on the vulnerable groups. It is also worthy to note that if public health responses exclude the marginalized “voices,” there is a potential threat of reversal in any hard-won progress and efforts in addressing such public health crisis.^7,8^ This highlights the need for African governments, national health authorities, and other stakeholders to lead the path in ensuring inclusive responses to public health emergencies.

Sex workers in some African countries (e.g., Nigeria, Uganda, and Botswana) are excluded from the government’s safety nets in response to COVID-19.^7^ This forced some sex workers back to work amid the lockdown imposed by the national health authorities in the early days of the pandemic. Lockdown policies in many African countries and globally have significantly affected access to antiretroviral drugs and care services among people living with HIV. People living with disabilities have also been significantly affected by uniform response activities in many African countries.^10^ According to a rapid virtual audit of pandemic-related press briefings and press conferences issued by governments and international organizations, only 54% of sub-Saharan African countries have a sign language interpreter present in COVID-19 press briefings and conferences.^11^ COVID-19 has already caused interruptions in vaccination schedules in many African countries, further putting many children at risk of vaccine-preventable diseases. It has also weakened public health responses to ailments such as malaria and meningitis outbreaks and reduced access to maternal and reproductive health services, further discriminately affecting at-risk groups. COVID-19 also highlights the vulnerability of healthcare professionals to the impact of public health emergencies and how they can be discriminately affected. This vulnerability is compounded by limited access to protective equipment and stigmatization, which impacts on their mental health, as reported in some African countries.^5^

Some researchers have also reported how people who use drugs are left out of the COVID-19 responses in some African countries, for example, Kenya and Nigeria, with the potential threat of affecting HIV response and access to healthcare services among this group.^8,12^ COVID-19 has also been reported to exacerbate long-standing problems in Africa’s prisons and how precautionary measures, such as handwashing and physical distancing to reduce disease transmission among the inmates, are challenging to observe because of poor infrastructure.^13^ A report from Kenya, Tanzania, and Uganda, the regions with consistently high maternal and neonatal mortality rates, also revealed that the COVID-19 pandemic has grossly affected antenatal care services.^14^ The disruption of these services, if not addressed, may affect the achievement of Sustainable Development Goal 3 Target 1, which aims to reduce the global maternal mortality ratio to less than 70 per 100,000 live births by 2030. The noted disruption in healthcare services in Africa is also affecting older adults with chronic diseases in the region.^15^ Access to healthcare services among people living with chronic diseases,^16^ people with lower socioeconomic status, homeless people, migrants, minorities, and other vulnerable groups is discriminately affected before the pandemic, and the urgent need to address the COVID-19 pandemic has exacerbated this challenge.

Although some efforts have been made in Africa toward ensuring an inclusive response, more efforts still need to be carried out. For instance, Ethiopia is translating COVID-19 health information into local languages^17^ and the country plans to make communication materials accessible to those with seeing, learning, and hearing difficulties, as well as people living with mental illness.^10^ It has also been reported that South Africa has granted $10.6 million aid to assist small, medium, and microenterprises, with people living with disabilities and women prioritized.^10^ In Nigeria, a faith-based organization is helping with sign language interpretation of COVID-19 health information.^10^ Africa’s response to public health emergencies needs to recognize that for every marginalized/vulnerable group, it is important that any health intervention designed to meet their needs must be carried out in a way that surmounts any barriers to accessing health care.

For Africa’s public health response to be more inclusive, there is a need to be more strategic and proactive in reaching out to specific groups to identify and address their peculiar needs (Table 1). An inclusive health approach needs action and not just mere inclusion in policy documents. The approach will not only foster solidarity, health equity, and effective community response to public health interventions and emergencies, it is also in line with the sixth element of the Universal Health Coverage movement—“Move Together”—which involves setting up diverse multi-stakeholder strategies aimed at engaging and involving everyone without discrimination. The significance of an inclusive, dynamic, multi-stakeholder response remains critical in the context of COVID-19 and other public health emergencies. A commitment to an inclusive health response implies that response activities will be compassionate and sensitive to all. The aftermath of a noninclusive public health response is expensive. For instance, if marginalized or vulnerable groups with poor access to healthcare services experience COVID-19–related symptoms, they may delay or even forgo being tested and may consequently turn to medical care only in late stages, resulting in worse outcomes. This may put their families at risk, facilitating community spread of the virus. Furthermore, African governments need to start devising means and strategies to ensure inclusive access to COVID-19 vaccine when it becomes available.^18^

**Table 1:** Specific health needs of some vulnerable groups amid the COVID-19 pandemic and recommendations

Vulnerable group	Specific health needs in the context of COVID-19	Recommendations
Older adults	Access to personal protective equipment (PPE)	Use telemedicine and other remote consultation strategies
	Social and mental health support	Integrate older adults’ care into primary healthcare systems
	Clear risk communication language	Use geriatrics differentiated healthcare services
	Undisrupted access to medicines and healthcare services, including COVID-19 testing	Leverage community health workers for nonspecialized healthcare support
Pregnant women	Antenatal care services including medications and specialist consultation	Devise locally compatible strategies to ensure uninterrupted antenatal care services
	Access to PPE	
	Social and mental health support	
Women and girls	Access to sexual and reproductive care services including family planning	Ensure access to sexual and reproductive health services, keeping clinics open and/or telehealth
	Access to menstrual hygiene care and products	Ensure access to contraceptives and other essential medicines
	Mental health and social care support to those facing violence and abuse	
People living with disabilities	Health needs associated with the impairment must be prioritized	Leverage community health workers for nonspecialized healthcare support
	Information on COVID-19 in accessible formats	Inclusive engagement for people living with disabilities
	Access to PPE	
	Social and mental health support	
People living with mental health disorders	Access to antipsychotic medications and psychosocial support should be prioritized	Use telemedicine and other remote consultation strategies
People who use drugs	Access to harm reduction services including needle exchange and opioid substitution therapy	Work with drug use advocacy groups and civil society organizations to ensure access to health services
	Access to care and prevention services for HIV, hepatitis, COVID-19, and sexually transmitted diseases	Decriminalization of drug use and avoidance of discrimination and stigmatization of people who use drugs
	Social and mental health support	
	Rehabilitation and drug treatment centers must be prioritized	
People living with HIV	Sustained access to HIV drugs and care services	Strengthening of HIV differentiated care services
	Social and mental health support	Decentralization of HIV care and use of telemedicine
	COVID-19 testing	Work with HIV advocacy groups to ensure access to health services
	Treatment and care services for tuberculosis and other opportunistic infections must be prioritized	
	Access to condoms and PPE	
People living with chronic diseases, for example, diabetes	Access to medicines and PPE	Use telemedicine and other remote consultation strategies
	Clear risk communication language	
	Social and mental health support	
Sex workers, men who have sex with men, and transgender people	Access to HIV care and prevention services	Work with the relevant advocacy groups and civil society organizations to ensure access to health services
	Access to hepatitis, COVID-19, and sexually transmitted diseases testing	Decriminalization and avoidance of discrimination and stigmatization of vulnerable groups should be prioritized to ensure access to health services
	Access to condoms and PPE	
	Social and mental health support	
People in prisons and other closed settings	Prison-based harm reduction services	Ensure that the right to health among inmates is not infringed on
	Access to condoms and PPE	Direct investment to enable African prisons to cope with public health emergencies
	Structures that allow for physical distancing and proper ventilation	
	Access to clean water and soap	
	COVID-19 testing	

## CONCLUSION

Africa’s COVID-19 and other health emergencies response strategies must be inclusive of marginalized and vulnerable groups to ensure they maintain respect for dignity, human rights, and fundamental freedoms and avoid widening already existing health and social disparities. This reinforces the need to ensure full participation of the communities affected and relevant civil society organizations in preparedness and response planning, including maintaining the commitment to universal health coverage and ensuring that the most disadvantaged groups are not forgotten amid the urgent need to respond to any public health emergencies. Strengthening the healthcare systems of African countries through increased political will, increased funding to health care, collaboration and cooperation among stakeholders, and effective leadership remains essential in ensuring inclusive responses to health emergencies.
